# Supplementary material for: Model-based standardization using multiple imputation
Source: BMC Med Res Methodol. 2024 Feb 10;24:32. doi: 10.1186/s12874-024-02157-x (PMC10858574; doi:10.1186/s12874-024-02157-x)
Supplement: Supplementary file 1 — Additional file 1. [file 12874_2024_2157_MOESM1_ESM.pdf]

Supplementary material of “Model-based standardization using multiple imputation”

Antonio Remiro-Azócar[1], Anna Heath[2,3,4], and Gianluca Baio[4]

[1]Statistics and Data Insights, Bayer plc
[2]Child Heath Evaluative Sciences, The Hospital for Sick Children
[3]Dalla Lana School of Public Health, University of Toronto
[4]Department of Statistical Science, University College London

Example code

Example `R` code implementing the standard version of model-based standardization and multiple imputation marginalization (MIM) is provided below. The code and data are available at `https://github.com/remiroazocar/MIM` in the `Example` subdirectory. Full code for the simulation study is available in the online repository.

Standard model-based standardization

```
library("boot") # for non-parametric bootstrap

ipd.index <- read.csv("index_IPD.csv") # load patient-level dataset of
    comparative index study
ipd.target <- read.csv("target_IPD.csv") # load target covariate individual
    -level dataset

set.seed(555) # set seed for reproducibility

# settings
resamples <- 1000 # number of resamples in non-parametric bootstrap

# function to be bootstrapped
gcomp.ml <- function(data, indices) {
  index.dat = data[indices,]
  # logistic outcome model fitted to index RCT subject-level data using
      maximum likelihood estimation
  outcome.model <- glm(y~(trt*x1)+(trt*x2), data=index.dat, family=binomial
      )
  # "counterfactual" target datasets ("target" covariates assumed fixed)
  target.t1 <- target.t0 <- ipd.target
  # intervene on assigned treatment
  target.t1$trt <- 1 # target dataset where everyone is assigned active
      treatment
```

```
  target.t0$trt <- 0 # target dataset where everyone is assigned control
  # predict potential individual-level event probabilities, conditional on
     treatment and covariates
  hat.mu.1.i <- predict(outcome.model, type="response", newdata=target.t1)
  hat.mu.0.i <- predict(outcome.model, type="response", newdata=target.t0)
  # marginal mean probability predictions under each treatment
  hat.mu.1 <- mean(hat.mu.1.i)
  hat.mu.0 <- mean(hat.mu.0.i)
  # transform from probability to linear predictor scale to estimate
     marginal log odds ratio
  hat.Delta <- log(hat.mu.1/(1-hat.mu.1)) - log(hat.mu.0/(1-hat.mu.0))
  # hat.Delta <- qlogis(hat.mu.1) - qlogis(hat.mu.0)
  return(hat.Delta)
}
# non-parametric bootstrap
boot.object <- boot::boot(data=ipd.index, statistic=gcomp.ml, R=resamples)
# bootstrap mean of marginal log odds ratio estimate
hat.Delta <- mean(boot.object$t)
# bootstrap variance of marginal log odds ratio estimate
hat.var.Delta <- var(boot.object$t)
# Interval estimates derived from the relevant percentiles across the
   bootstrap resamples
conf.ints <- quantile(boot.object$t, probs = c(.025, .975), type = 6)
lci.Delta <- conf.ints[1] # lower interval bound
uci.Delta <- conf.ints[2] # upper interval bound
```

MIM

```
library("rstanarm") # for the synthesis stage

ipd.index <- read.csv("index_IPD.csv") # load patient-level dataset of
   comparative index study
ipd.target <- read.csv("target_IPD.csv") # load target covariate individual
   -level dataset

set.seed(555) # set seed for reproducibility

# settings
M <- 1000 # number of syntheses/imputations used in analysis stage (high
   for low Monte Carlo error)
n.chains <- 2 # number of Markov chains for MCMC sampler in synthesis stage
warmup <- 2000 # number of discarded warmup/burn-in iterations per chain
   for MCMC sampler
iters <- 4000 # number of total iterations per chain for MCMC sampler (
   including warmup)

## SYNTHESIS STAGE ##
# first-stage logistic regression model fitted to index RCT using MCMC (
   Stan)
outcome.model <- stan_glm(y~(trt*x1)+(trt*x2), data=ipd.index, family=
   binomial, algorithm="sampling",
                          iter=iters, warmup=warmup, chains=n.chains,
                          # thin to use M independent samples in analysis
                             stage
```

```
                          thin=(n.chains*(iters-warmup))/M)
# create augmented target dataset
target.t1 <- target.t0 <- ipd.target
target.t1$trt <- 1 # assign active treatment in synthesis
target.t0$trt <- 0 # assign control in synthesis
aug.target <- rbind(target.t0, target.t1)
# complete syntheses by drawing binary outcomes from their posterior
    predictive distribution
y.star <- posterior_predict(outcome.model, newdata=aug.target)

## ANALYSIS STAGE ##
# fit second-stage regression to each synthesis using maximum-likelihood
    estimation
reg2.fits <- lapply(1:M, function(m) glm(y.star[m,]~trt, data=aug.target,
    family=binomial))
# treatment effect point estimates given by treatment coefficient for each
    synthesis
hats.delta <- unlist(lapply(reg2.fits, function(fit) coef(fit)["trt"][[1]])
    )
# point estimates for the variance in each synthesis
hats.v <- unlist(lapply(reg2.fits, function(fit) vcov(fit)["trt", "trt"]))
# quantities originally defined by Rubin (1987) for multiple imputation
bar.delta <- mean(hats.delta) # average of treatment effect point estimates
bar.v <- mean(hats.v) # "within" variance (average of variance point
    estimates)
b <- var(hats.delta) # "between" variance (sample variance of point
    estimates)
# pooling: average of point estimates is marginal log odds ratio
hat.Delta <- bar.delta
# pooling: use combining rules to estimate the variance
hat.var.Delta <- (1+(1/M))*b-bar.v
# Wald-type interval estimates constructed using t-distribution with nu
    degrees of freedom
nu <- (M-1)*(1+bar.v/((1+1/M)*b))^2
lci.Delta <- hat.Delta + qt(0.025, df=nu)*sqrt(hat.var.Delta)
uci.Delta <- hat.Delta + qt(0.975, df=nu)*sqrt(hat.var.Delta)
```

MIM within the index study and with missing outcomes

In the main text, we have assumed that the comparative index study has no missing values and used MIM to standardize over the covariate distribution of an external target. We will now show, helped by a simple simulation study, that MIM can perform standardization over the empirical covariate distribution observed for the index study. We will also assume that there are some missing subject-level outcomes in the index study, thereby illustrating how MIM can handle this issue.

Methods

There is no longer an external target covariate dataset, or rather the empirical covariate distribution of the index study makes up the target. The target marginal average

treatment effect estimand, in terms of potential outcomes, is now $g\left(E\left(Y^1 \mid S=1\right)\right) - g\left(E\left(Y^0 \mid S=1\right)\right)$, where $S=1$ denotes the index study.

As per the main text, individual-level data $\mathcal{D} = (\boldsymbol{x}, \boldsymbol{t}, \boldsymbol{y})$ are available for a comparative index study, where $\boldsymbol{x}$ is an $N \times K$ matrix of baseline covariates, with N denoting the number of subjects in the study, K denoting the number of baseline covariates, and with each subject $n = 1, 2, \ldots, N$ contributing a row vector $\boldsymbol{x}_n = (x_{n,1}, x_{n,2}, \ldots, x_{n,K})$ of K covariates. Again, $\boldsymbol{t} = (t_1, t_2, \ldots, t_N)$ denotes a binary treatment indicator vector and $\boldsymbol{y} = (y_1, y_2, \ldots, y_N)$ denotes a vector of clinical outcomes. In this case, there are some missing outcomes such that $\boldsymbol{y} = (\boldsymbol{y}_{\text{obs}}\ \boldsymbol{y}_{\text{mis}})$, where $\boldsymbol{y}_{\text{obs}}$ is a vector of observed outcomes and $\boldsymbol{y}_{\text{mis}}$ denotes the outcomes that have not been observed.

The first-stage covariate-adjusted regression is fitted to the units of the index study with observed outcomes. In this case, the "data augmentation" step vertically concatenates a copy of the index study covariates to the original $\boldsymbol{x}$, such that the concatenation is denoted $\boldsymbol{x^*} = \left[\begin{smallmatrix}\boldsymbol{x}\\ \boldsymbol{x}\end{smallmatrix}\right]$ and has $N^* = (2 \times N)$ rows and K columns. When augmenting the original covariate dataset, the actual treatment assignment is discarded, with the original $j = 1, 2, \ldots, N$ rows assigned the treatment value $t_j^* = 1$ and the appended $j = (N+1), (N+2) \ldots, N^*$ rows assigned the treatment value $t_j^* = 0$. The treatment indicator vector in the augmented dataset is denoted $\boldsymbol{t^*} = (t_1^*, t_2^* \ldots t_{N^*}^*)$.

As per the main text, the synthesis stage, i.e. the estimation of the first-stage regression and the outcome prediction step, is implemented within a single Bayesian computation module using Markov chain Monte Carlo (MCMC) sampling. Having fitted the first-stage covariate-adjusted regression to the index study subjects with observed outcomes, this is used to generate predicted "hypothetical" outcomes, based on the posterior predictive distribution of observed outcomes. To fill in the synthetic datasets, predicted outcomes are generated simultaneously for both the subjects that have missing and non-missing actual outcome values.

As per the main text, one iterates over the L converged draws of the MCMC algorithm to generate M synthetic datasets $\{\mathcal{D}^* = \mathcal{D}^{*(m)} : m = 1, 2 \ldots, M\}$, where $\mathcal{D}^{*(m)} = \left(\boldsymbol{x^*}, \boldsymbol{t^*}, \boldsymbol{y}^{*(m)}\right)$, and thinning is applied so that only $M \leq L$ synthetic datasets are retained for the analysis stage. Covariates $\boldsymbol{x^*}$ and treatment $\boldsymbol{t^*}$ are fixed across all the synthetic datasets, and each synthetic dataset is completed by drawing a vector of outcomes $\boldsymbol{y}^{*(m)} = \left(y_1^{*(m)}, y_2^{*(m)}, \ldots, y_{N^*}^{*(m)}\right)$ of size N^* from their posterior predictive distribution. Table S1 illustrates the structure of each synthetic dataset. The analysis stage would proceed just as described in the main text.

The described scheme has an important limitation, which is that it relies on the missingness pattern for the index study subject-level outcomes being monotone. While such assumption may have some plausibility where missing outcomes arise due to dropout, it may not be a realistic assumption.

The scheme could incorporate additional models to impute missing subject-level covariate and treatment values in the index study, in each MCMC iteration of the synthesis stage, under a missing-at-random assumption and as long as such models are congenial with the covariate-adjusted model specified for standardization and outcome imputation. This would be feasible under a Bayesian joint modelling framework and could be programmed using dedicated MCMC software such as `Stan`.[1]

Table S1: An example of the structure of the m-th synthetic dataset, created in the data synthesis stage of MIM. The actual outcome ($\boldsymbol{y}$) would not be part of the synthetic dataset but is displayed for illustrative purposes. In this example, $N = 7$ ($N^* = 14$), $K = 3$, and there are 5 observed outcomes and 2 missing outcomes in the original index study. Prior to generating the predicted outcomes, a copy of the original index study covariates has been assigned the treatment value zero and has been vertically concatenated to the original $\boldsymbol{x}$, assigned the treatment value one.

Covariates ($\boldsymbol{x}^*$)			Treatment ($\boldsymbol{t}^*$)	Actual outcome ($\boldsymbol{y}$)	Predicted outcome $\left(\boldsymbol{y}^{*(m)}\right)$
$x_{1,1}$	$x_{1,2}$	$x_{1,3}$	1	y_1	$y_1^{*(m)}$
$x_{2,1}$	$x_{2,2}$	$x_{2,3}$	1	y_2	$y_2^{*(m)}$
$x_{3,1}$	$x_{3,2}$	$x_{3,3}$	1	y_3	$y_3^{*(m)}$
$x_{4,1}$	$x_{4,2}$	$x_{4,3}$	1	y_4	$y_4^{*(m)}$
$x_{5,1}$	$x_{5,2}$	$x_{5,3}$	1	y_5	$y_5^{*(m)}$
$x_{6,1}$	$x_{6,2}$	$x_{6,3}$	1	NA	$y_6^{*(m)}$
$x_{7,1}$	$x_{7,2}$	$x_{7,3}$	1	NA	$y_7^{*(m)}$
$x_{1,1}$	$x_{1,2}$	$x_{1,3}$	0	y_1	$y_8^{*(m)}$
$x_{2,1}$	$x_{2,2}$	$x_{2,3}$	0	y_2	$y_9^{*(m)}$
$x_{3,1}$	$x_{3,2}$	$x_{3,3}$	0	y_3	$y_{10}^{*(m)}$
$x_{4,1}$	$x_{4,2}$	$x_{4,3}$	0	y_4	$y_{11}^{*(m)}$
$x_{5,1}$	$x_{5,2}$	$x_{5,3}$	0	y_5	$y_{12}^{*(m)}$
$x_{6,1}$	$x_{6,2}$	$x_{6,3}$	0	NA	$y_{13}^{*(m)}$
$x_{7,1}$	$x_{7,2}$	$x_{7,3}$	0	NA	$y_{14}^{*(m)}$

Simulation study

A simple simulation study investigates the use of MIM to standardize over the empirical covariate distribution of the index study. The data-generating mechanisms are almost identical to those described in the main text, but no external target covariate dataset is generated; the covariate distribution observed for the index study is assumed to be the target. In addition, some subject-level outcomes in the index study are assumed to be missing completely at random. We vary the missingness probability $\pi \in \{0.1, 0.2, 0.3, 0.4\}$; there are four simulation scenarios, each corresponding to a different missingness probability. We consider the index study to be a randomized trial of size $N = 750$, with a 1:1 active treatment vs. control allocation ratio.

The target estimand is the true marginal log odds ratio for active treatment versus control in the index study covariate distribution. This is determined following the simulation-based procedure described in the main text. In the index study, the true marginal outcome probabilities for active treatment and control are 0.46 and 0.66, respectively, resulting in a true marginal log odds ratio of -0.84.

As per the main text, each simulated dataset is analyzed using MIM, following the procedure described earlier in this section. The first-stage logistic regression is correctly

specified and the synthesis stage is performed using the `R` package `rstanarm`.[2] We apply the default normally-distributed "weakly informative" priors for the regression coefficients, two Markov chains with 4,000 iterations per chain and 2,000 "burn-in" iterations per chain, and thinning every 4 iterations to use a total of $M = (2000 \times 2)/4 = 1000$ syntheses of size $N^* = 2 \times 750 = 1500$ in the analysis stage. The analysis stage is identical to that of the simulation study in the main text: second-stage logistic regressions are fitted using maximum-likelihood estimation, point estimates and variances are pooled using the combining rules, and Wald-type 95% confidence intervals are estimated using t-distributions with $\nu_f = (M-1)\left(1+\bar{v}/\left((1+1/M)\,b\right)\right)^2$degrees of freedom. Variance estimates are never negative under any missingness scenario.

We simulate 1,000 data replicates per scenario, computing the following performance metrics: (1) bias; (2) empirical standard error (ESE); (3) mean square error (MSE); and (4) empirical coverage rate of the 95% interval estimates. Monte Carlo standard errors (MCSEs) are reported for each performance measure to quantify the simulation uncertainty.

The simulations and analyses have been performed using `R` software version 4.1.1.[3] The file required to run the simulations is available at `http://github.com/remiroazocar/MIM/appendix.R`.

Performance metrics for MIM across the four missingness scenarios are reported in Table S2, with MCSEs presented in parentheses alongside the corresponding performance measure. The MIM estimator has almost negligible bias across the scenarios. As would be expected, the ESE and MSE have greater magnitude – precision and efficiency decrease – as the missingness proportion increases. The interval estimates seem close to the nominal 0.95 rate, suggesting that uncertainty quantification is adequate, but perhaps exhibiting some slight undercoverage (e.g. for $\pi = 0.1$).

Table S2: Performance measures with MCSEs for MIM across scenarios with different missingness proportions.

Missingness (π)	Bias	ESE	MSE	Coverage
0.1	0.004 (0.005)	0.159 (0.004)	0.025 (0.001)	0.931 (0.008)
0.2	-0.003 (0.005)	0.163 (0.004)	0.026 (0.001)	0.942 (0.007)
0.3	-0.010 (0.006)	0.179 (0.004)	0.032 (0.002)	0.937 (0.008)
0.4	-0.013 (0.006)	0.190 (0.004)	0.036 (0.001)	0.946 (0.007)

Extension of MIM to target multi-component estimands

The inferential framework for pooling outlined in the main text can be extended to scenarios involving correlated outcomes and non-scalar estimands with multiple components. This involves a multivariate outcome model (i.e. with multiple dependent variables) and the combination of correlated treatment effects corresponding to multiple outcomes.

With non-scalar estimands, the pooling stage must propagate the covariance or correlation structure of treatment effects through the analysis. Consequently, the inferential

framework for pooling outlined in the main text has been extended by Reiter.[4] The column vector of treatment effect point estimates for the m-th synthesis is denoted $\hat{\boldsymbol{\xi}}^{(m)}$ and has $D \geq 2$ components. The estimated $D \times D$ covariance matrix of treatment effect estimates for the m-th synthesis is denoted $\hat{\boldsymbol{v}}^{(m)}$. Analogous to Equations 4, 5 and 6 in the main text, the following multivariate quantities are required for inference:

$$\bar{\boldsymbol{\xi}} = \sum_{m=1}^{M} \hat{\boldsymbol{\xi}}^{(m)}/M, \tag{1}$$

$$\bar{\boldsymbol{v}} = \sum_{m=1}^{M} \hat{\boldsymbol{v}}^{(m)}/M, \tag{2}$$

$$\boldsymbol{b} = \sum_{m=1}^{M} (\hat{\boldsymbol{\xi}}^{(m)} - \bar{\boldsymbol{\xi}})(\hat{\boldsymbol{\xi}}^{(m)} - \bar{\boldsymbol{\xi}})^{\top}/(M-1). \tag{3}$$

Here, $\bar{\boldsymbol{\xi}}$ is a vector of size D of treatment effect point estimates across the M syntheses, $\bar{\boldsymbol{v}}$ is a $D \times D$ matrix of the average estimated covariance matrices, and $\boldsymbol{b}$ is the $D \times D$ sample covariance matrix of the treatment effect point estimates.

The target estimands for inference are the marginal treatment effects in the target covariate distribution, denoted by vector $\boldsymbol{\Xi}$. The posterior distribution $p(\boldsymbol{\Xi} \mid \boldsymbol{y}^{*}, \boldsymbol{t}^{*})$ is assumed to be approximately multivariate normal and is constructed as:

$$p(\boldsymbol{\Xi} \mid \boldsymbol{y}^{*}, \boldsymbol{t}^{*}) = \int_{\boldsymbol{\mu}_{\boldsymbol{\Xi}}, \boldsymbol{\Sigma}_{\boldsymbol{\Xi}}} p(\boldsymbol{\Xi} \mid \boldsymbol{\mu}_{\boldsymbol{\Xi}}, \boldsymbol{\Sigma}_{\boldsymbol{\Xi}}) p(\boldsymbol{\mu}_{\boldsymbol{\Xi}}, \boldsymbol{\Sigma}_{\boldsymbol{\Xi}} \mid \boldsymbol{y}^{*}, \boldsymbol{t}^{*}) d(\boldsymbol{\mu}_{\boldsymbol{\Xi}}, \boldsymbol{\Sigma}_{\boldsymbol{\Xi}}), \tag{4}$$

with the posterior density parametrized by two moments: a vector of means $\boldsymbol{\mu}_{\boldsymbol{\Xi}}$ and a $J \times J$ covariance matrix $\boldsymbol{\Sigma}_{\boldsymbol{\Xi}}$. After deriving the quantities in Equations 1, 2, 3, the posterior in Equation 4 is approximated by the following distributions (by analogy to Equations 7, 8 and 9 in the main text):

$$p(\boldsymbol{\mu}_{\boldsymbol{\Xi}} \mid \boldsymbol{y}^{*}, \boldsymbol{t}^{*}) \sim \mathrm{N}(\bar{\boldsymbol{\xi}}, \bar{\boldsymbol{v}}/M), \tag{5}$$

$$p\left((M-1)\boldsymbol{b}/(\boldsymbol{\Sigma}_{\boldsymbol{\Xi}} + \bar{\boldsymbol{v}}) \mid \boldsymbol{y}^{*}, \boldsymbol{t}^{*}\right) \sim \mathrm{Wishart}_{M-1}, \tag{6}$$

$$p(\boldsymbol{\Xi} \mid \boldsymbol{\mu}_{\boldsymbol{\Xi}}, \boldsymbol{\Sigma}_{\boldsymbol{\Xi}}) \sim t_{M-1}\left(\boldsymbol{\mu}_{\boldsymbol{\Xi}}, (1+1/M)\boldsymbol{\Sigma}_{\boldsymbol{\Xi}}\right). \tag{7}$$

Note that the division in the left-hand side of Equation 6 is an element-wise (Hadamard) division. One can approximate the integral of the posterior in Equation 7 with respect to the posteriors in Equations 5 and 6 via simulation. However, it is considerably simpler to use a multivariate normal approximation to the posterior density in Equation 4, with means $\bar{\boldsymbol{\xi}}$ and covariance $(1+1/M)\boldsymbol{b} - \bar{\boldsymbol{v}}$, such that the sampling distribution in Equation 7 is normal. This yields the following combining rules,[4] used to derive point estimates, $\hat{\boldsymbol{\Xi}}$ and $\widehat{\boldsymbol{Cov}}(\hat{\boldsymbol{\Xi}})$, for the marginal treatment effects in the target covariate distribution and their covariance matrix, respectively:

$$\hat{\boldsymbol{\Xi}} = \bar{\boldsymbol{\xi}},$$

$$\widehat{\boldsymbol{Cov}}(\hat{\boldsymbol{\Xi}}) = (1+1/M)\boldsymbol{b} - \bar{\boldsymbol{v}}.$$

References

[1] Carpenter B, Gelman A, Hoffman MD, Lee D, Goodrich B, Betancourt M, et al. Stan: A probabilistic programming language. Journal of statistical software. 2017;76(1).

[2] Goodrich B, Gabry J, Ali I, Brilleman S. rstanarm: Bayesian applied regression modeling via Stan. R package version. 2020;2(1).

[3] Team RC, et al. R: A language and environment for statistical computing. 2013.

[4] Reiter JP. Significance tests for multi-component estimands from multiply imputed, synthetic microdata. Journal of Statistical Planning and Inference. 2005;131(2):365-77.
